# Rise of the natural red pigment ‘prodigiosin’ as an immunomodulator in cancer

**DOI:** 10.1186/s12935-022-02815-4

**Published:** 2022-12-28

**Authors:** Mohammed Moustapha Anwar, Chris Albanese, Nadia M. Hamdy, Ahmed S. Sultan

**Affiliations:** 1 Department of Biotechnology, Institute of Graduate Studies and Research (IGSR), Alexandria University, Alexandria, Egypt; 2 Oncology and Radiology Departments, Lombardi Comprehensive Cancer Center, Washington, D.C. USA; 3 Department of Biochemistry, Ain Shams Faculty of Pharmacy, Cairo, Egypt; 4 Biochemistry Department, Faculty of Science, Alexandria University, Alexandria, Egypt

**Keywords:** Prodigiosin, TME, NK Cells, T Cells, B Cells, TAMs, TADCs, MDSCs

## Abstract

Cancer is a heterogeneous disease with multifaceted drug resistance mechanisms (e.g., tumour microenvironment [TME], tumour heterogeneity, and immune evasion). Natural products are interesting repository of bioactive molecules, especially those with anticancer activities. Prodigiosin, a red pigment produced by *Serratia marcescens*, possesses inherent anticancer characteristics, showing interesting antitumour activities in different cancers (e.g., breast, gastric) with low or without harmful effects on normal cells. The present review discusses the potential role of prodigiosin in modulating and reprogramming the metabolism of the various immune cells in the TME, such as T and B lymphocytes, tumour-associated macrophages (TAMs), natural killer (NK) cells, and tumour-associated dendritic cells (TADCs), and myeloid-derived suppressor cells (MDSCs) which in turn might introduce as an immunomodulator in cancer therapy.

## Introduction

Cancer is the leading cause of death in 135 countries according to the World Health Organisation (WHO) global health estimates in 2019 [1]. The Global Burden of Cancer (GLOBOCAN) also reported ~ 19 million new cancer cases in 2020 that are anticipated to increase by 47% (~ 28 million) in 2040 [2]. Understanding tumour biology has facilitated the development of targeted therapies; however, tumours display multidrug resistance (MDR) as a significant clinical burden due to heterogeneity [3–6]. Natural bioactive compounds from various sources (e.g., plants, microbes) have emerged as immunomodulators in diseases, such as diabetes, cardiovascular diseases (CVDs), inflammation, and cancer [7–9].

Research deems the use of natural compounds as ‘immunomodulators’ alongside the advanced understanding of the complex interactions between cancer and the immune system [10]. Immunomodulators boost the immune defences against threats (e.g., infections) or quench the abnormal immune response in immune-related disorders [11]. Natural compounds are proven to affect immune cells and to enhance anticancer immune responses in vitro and in patients. For example, berries—which contain multiple chemopreventive compounds—enhance the function of natural killer (NK) cells and decrease the number of infiltrating neutrophils in colorectal cancer (CRC) [12–14]. Epigallocatechin gallate (EGCG), resveratrol, all-trans retinoic acid (ATRA), curcumin, polysaccharide K (PSK), β-glucans, and carotenoids are also immunomodulators (e.g., elevate NK cells and inhibit myeloid-derived suppressor cells [MDSCs]) [15–19]. Notably, bacteria-based cancer immunotherapy has lured attention thanks to its distinctive and ample components, mechanisms, and benefits to stimulate the host immunity against cancer [20].

**Fig. 1:**
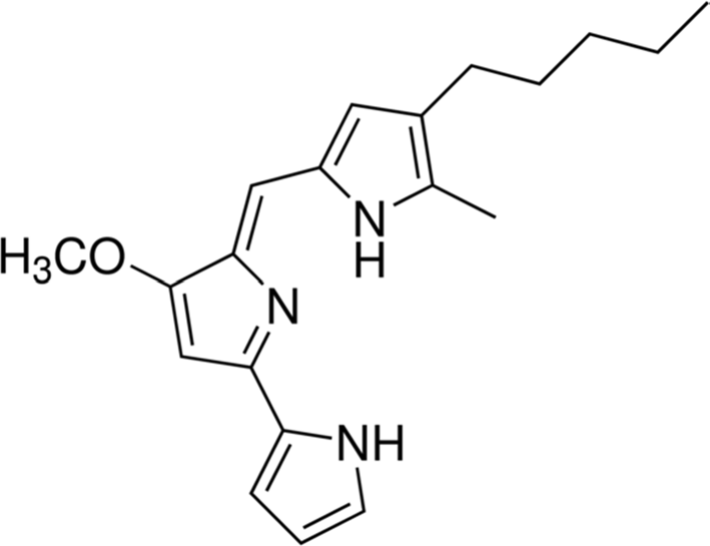
Structure of prodigiosin (2-methyl-3-pentyl-6-methoxyprodiginine)

**Fig. 2:**
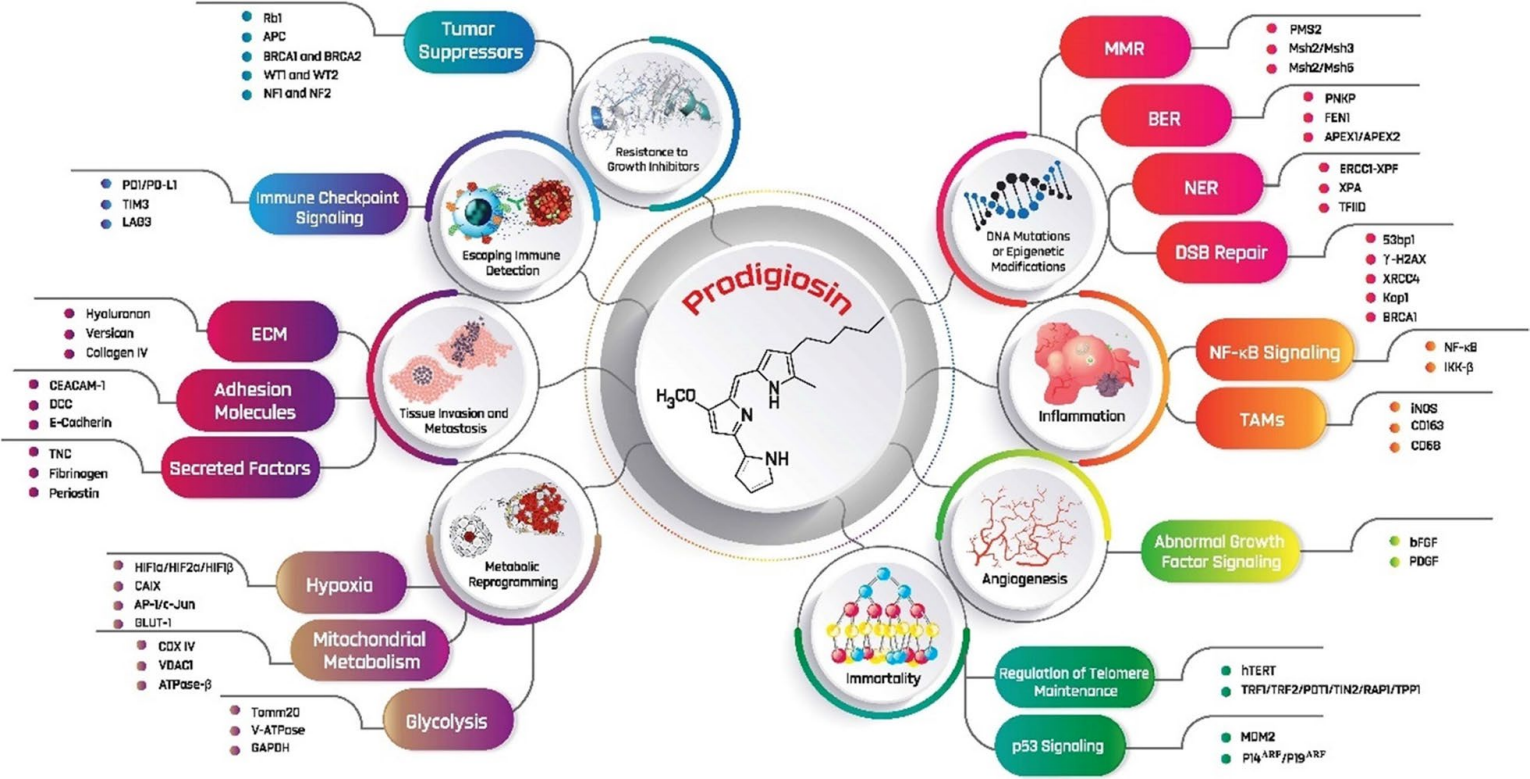
Cancer biomarkers where prodigiosin may exert anticancer actions. APC, adenomatous polyposis coli; APEX, AP DNA endonuclease; ATPase-β, adenosine triphosphatase-beta; BER, base excision repair; bFGF, basic fibroblast growth factor; BRCA1, breast cancer 1; BRCA2, breast cancer 2; CAIX, carbonic anhydrase IX; CD68, cluster of differentiation 68; CD163, cluster of differentiation 163; CEACAM-1, CEA cell adhesion molecule-1; COX IV, cytochrome C oxidase subunit IV; DCC, deleted in colorectal carcinoma; DSB, double-strand break; ECM, extracellular matrix; ERCC1-XPF, excision repair cross complementing protein 1-xeroderma pigmentosum group F; FEN1, flap endonuclease 1; GAPDH, glyceraldehyde-3-phosphate dehydrogenase; GLUT-1, glucose transporter protein type 1; HIF1α, hypoxia-inducible factor 1-alpha; HIF1β, hypoxia-inducible factor 1-beta; hTERT, human telomerase reverse transcriptase; IKK-β, inhibitor of nuclear factor kappa-B kinase; iNOS, inducible nitric oxide synthase; Kap1, kruppel-associated box (KRAB)-associated protein 1; LAG3, lymphocyte-activation gene 3; MDM2, mouse double minute 2; MMR, mismatch repair; Msh, MutS homolog 2; NER, nucleotide excision repair; NF-κB, nuclear factor kappa B; NF1, neurofibromatosis 1; PD1/PD-L1, programmed cell death 1/programmed death ligand 1; PDGF, platelet-derived growth factor; PMS2, PMS1 homolog 2; PNKP, polynucleotide kinase 3'-phosphatase; POT1, protection of telomeres 1; PTEN, Phosphatase and TENsin; V-ATPase, vacuolar proton-translocating adenosine triphosphatase; RAP1, repressor activator protein 1; Rb1, retinoblastoma protein 1; TAMs, tumor-associated macrophages; TFIID, transcription factor II D; TIN2, TRF1-interacting protein 2; TIM3, T-cell immunoglobulin mucin-3; Tomm20, translocase of outer mitochondrial membrane 20; TNC, tenascin C; TPP1, tripeptidyl peptidase 1; TRF, telomere restriction fragment; VDAC1, voltage-dependent anion-selective channel 1; WT1, Wilms' tumor 1; WT2, Wilms' tumor 2; XPA, xeroderma pigmentosum group A; XRCC4, X-ray repair cross complementing 4

Prodigiosin is a secondary metabolite anticancer red pigment that belongs to the “prodiginines” family, and is produced by the Gram-negative bacteria *Serratia marcescens* (Fig. 1) [21]. It inhibits the mammalian target of rapamycin (mTOR) pathway and angiogenesis, and induces cycle arrest and apoptosis in cancer cells with minimal or without observed cytotoxicity on healthy cells [22]. Inherent toxicity is one of the major issues with immunosuppressants that prompted researchers to use combined regimens, especially in oncology. Prodigiosin offers interesting possibilities for a combinatorial applications, acting synergistically with cyclosporin A and additively with rapamycin, confirming its distinctiveness and the potential for further development as immunosuppressants [23–25]. Of note, prodigiosin analogues have demonstrated a good safety profile without genotoxicity in clinical trials for the treatment of chronic lymphocytic leukaemia (CLL) [26]. Although prodigiosin is a well-established anticancer molecule (Table 1), its immunomodulating and metabolic reprogramming activities were not studied─data are only available for a related compound, prodigiosin 25-C [27–29]. Therefore, the current review discusses a compendium of possible immunomodulating and metabolic reprogramming activities of prodigiosin on specific immune cells and their cytokines in cancer. The present review also provides a comprehensive list of target biomarkers for prodigiosin in the tumour microenvironment (TME) (Fig. 2) [30–39].

**Table 1:** Evidence-based anticancer effects of prodigiosin

Cancer(s) type	Study model(s)	Anticancer effect(s)	Reference(s)
*Ovarian*	A2780RCIS (MRP1, 2 overexpressing cell line)	↓ Cell viability Acts regardless of the BCRP, MDR1, or MRP transporter	[40]
*Gastric*	EPG85-257RNOV EPG85-257RDB		
	HGT-1 cell line	↑ Apoptosis ↓ Cell viability Cell shrinkage Cell detachment from the culture substrate	[41]
*Glioblastoma*	U87MG cell line GBM8401 cell line	Stimulates stress markers of ER (e.g., BiP/GRP78, CHOP, and sXBP1) ↑ Autophagic cell death Activates the JNK pathway ↓ Decreasing the AKT/mTOR pathway ↑ Caspase 3 levels ↑ PARP cleavage ↑ LC3-II/LC3-I ↑ Bax/β-Actin ratio ↓ p62	[42]
*Neuroblastoma*	LAN-1 IMR-2 SK-N-AS SH-SY5Y	Uncouples the protons of the ETC to mitochondrial ATP synthase ↓ ATP production	[43]
*Colorectal cancer (CRC)*	HT-29 cell line	↓ G2/M ↑ Blockage in the G1 phase ↓ Number of viable cells ↓ Survivin mRNA levels ↑ Caspase 3 levels ↑ *Bax* mRNA levels ↑ *Bad* mRNA levels ↑ P53 protein levels ↓ *Bcl-2* mRNA levels	[44–46]
	DLD-1 cells SW-620 cells	↑ Apoptosis ↑ Caspases levels Acts irrespective of *p53* status (mutant or absent) PARP cleavage	[47]
	DLD-1 cells	↑ P53 protein levels ↑ Apoptosis ↑ Lysosomal pH	[46]
	WiDr cells	↑ Anticancer activity	[48]
	NRK normal cells Swiss-3T3 normal cells	No significant decrease in viable cells No apoptosis No toxicity	[47]
	HCT116 cells SW480 cells HT-29 cells N87 cells AGS cells LoVo cells Nude BALB/c male mice	Accumulation of LC3B-II and SQSTM ↓ Lysosomal activity by accumulating EGFP-LC3 puncta Triggers autophagy ↑ LC3-II/LC3-I ↑ Caspase 3 levels ↑ *in-vitro* sensitivity to 5-FU ↑ *in-vivo* 5-FU efficacy	[49]
	SW480 cells HCT116 cells DLD1 cells Athymic nu/nu mice	Restores *P53* Activates P73 Prevents formation of colonosphere irrespective of *p53* ↓ Viability of self-renewing 5-fluorouracil-resistant Aldefluor( +) CRCSCs ↓ Growth of xenograft tumours initiated with Aldefluor( +) cells without toxic effects and limits their tumourigenesis Activates a *p53*-responsive luciferase reporter in colonospheres ↓ Levels of the oncogenic N-terminally truncated isoform ΔNp73 in Aldefluor( +) cells ↑ Levels of the transcription factor c-Jun	[50]
	P53 mutant SW480 cells	Rescues a deficient *P53* pathway ↑ Antitumour effects via disruption of the mutant *P53*/*P73* complex and P73 upregulation	[51]
*Breast*	T47D cell line	No effect on cell cycle ↑ Signature ER stress markers (i.e., CHOP and GRP78) ↑ Caspase 3 levels ↑ Bax expression levels ↑ Bak expression levels ↓ Bcl-2 expression levels ↓ Survivin transcription levels ↑ Apoptosis ↓ RAD51 mRNA expression ↑ JNK signalling pathway ↑ P38 MAPK signalling pathway	[44, 52–55]
	MCF-7 cell line	↑ Signature ER stress markers (i.e., CHOP and GRP78) ↑ Apoptosis ↓ RAD51 mRNA expression ↑ JNK signalling pathway ↑ P38 MAPK signalling pathway Activates the IRE1–JNK pathway Activation of GSK3β Accumulation of P53 protein ↑ Bak expression levels ↑ Caspase 3 levels ↑ Caspase 7 levels ↓ Bcl-2 expression levels ↓ Survivin transcription levels ↑ Bax expression levels Activates NAG-1 Activates the PERK–eIF2α pathway ↑ P53 protein levels ↑ PUMA protein levels Arrests cell cycle at G_1_ phase	[52–58]
	MDA-MB-231 cell line	↓ *Bcl-2* transcription and expression levels ↓ Cell viability ↓ Proliferation ↓ Phosphorylated LRP6 ↓ Phosphorylated DVL2 ↑ Apoptosis ↑ Caspase 3 levels ↑ Caspase 8 levels ↑ Caspase 9 levels No effect on Raf-1 ↑ Bak expression levels ↓ Survivin transcription and expression levels ↓ *HSP90ɑ* mRNA and protein levels ↑ *Bax* mRNA levels ↓ RAD51 mRNA expression ↑ JNK signalling pathway ↑ P38 MAPK signalling pathway ↓ mTOR expression levels ↓ EGFR expression levels ↓ VEGF expression levels PARP cleavage Blocked Wnt/β-Catenin signalling ↓ CDK1 levels ↓ phosphorylated GSK3β ↓ β-catenin gene expression Supports normal breast cell proliferation or growth^*^ Prevents tumour locoregional recurrence in vivo^*^ Causes significant breast cancer cell death^*^	[53, 55, 56, 59–63]
	KPL-1 cell line MKL-F cell line	↑ Apoptosis ↑ Bak expression levels Activates caspase 3 ↓ Bcl-2 expression levels ↑ Bax expression levels	[53]
	MDA-MB-468 cell line	↓ Cell viability ↓ Proliferation ↑ Apoptosis Blocked Wnt/β-Catenin ssignalling ↓ Phosphorylated LRP6 ↓ Phosphorylated DVL2 ↓ phosphorylated GSK3β ↓ β-catenin gene expression	[60]
	MDA-MB-231 xenografts MMTV-Wnt1 transgenic mice	↓ Tumour progression ↓ Ser9 phosphorylated GSK3β ↓ Wnt/β-Catenin ssignalling ↓ CDK levels ↓ Phosphorylated LRP6 ↓ Phosphorylated and unphosphorylated DVL2 ↓ Active β-catenin	
*Haematopoietic*	Acute human T cell leukaemia cells (Jurkat clone E6-1) NSO myeloma cells HL-60 human promyelocytic leukaemia cells Human Burkitt lymphoma cells (Ramos)	↓ Number of viable cells ↑ Apoptosis Acts in absence of *p53* ↓ Vacuolar ATPase No significant toxicity, apoptosis, or decrease in normal cells	[64]
	Acute human T cell leukaemia cells (Jurkat clone E6-1)	↑ Phosphorylation of p38-MAPK	[65]
	Wt-p53Molt-4 cells (T-ALL)	↓ Survivin protein levels ↑ Caspase 3 levels ↑ accumulation of P53 Less uniform cells without membrane integrity ↓ Number of viable cells Diminishes metabolic activity ↓ Rate of proliferation	[66]
	CCRF-CEM cells	↓ Proliferation rate ↓ Viable cell number ↓ Survivin mRNA and protein levels ↓ MMP-9 mRNA and protein levels ↑ Caspase 3 ↑ Apoptosis	[67]
	B and T cells	↑ Apoptosis ↑ Caspase 3 levels ↑ Caspase 9 levels	[68]
*HCC*	HepG2 WiDr cells	Changes cellular morphology to apoptotic types Disrupts cell connections ↓ Cell proliferation ↓ Metabolic activity Activates caspase 3 ↓ survivin expression ↑ Apoptotic rate ↑ Anticancer activity	[48, 69]
*Pancreatic*	H8898 cell line	↓ Cell proliferation ↑ mitotic arrest ↑ ROS levels Cell death DNA fragmentation ↑ Apoptosis	[70]
*Lung*	Doxorubicin-sensitive A549 cell line Doxorubicin-resistant anti-Dox-A549 cell line	↑ Cytotoxicity ↑ Anticancer activity ↑ PARP cleavage ↑ Apoptosis Activates autophagy ↓ Autophagic inhibitor expression Activates non-PI3K-Class III/Beclin-1 inducer expression ↓ PI3K-p85/AKT/mTOR signalling pathways	[48, 71, 72]
	Doxorubicin-sensitive- and -resistant-bearing C57BL/6 mice	No acute toxicity ↓ Tumor cell accumulation around the trachea	
	A549 cell line HSAEC cells (i.e., an immortalised healthy cell line)	No cytotoxic effect on healthy cells ↓ Cell viability Changes morphology ↓ DNA replication ↑ Metabolic rewiring	[72]
	A549 cells CL1-5 cells H23 cells 293 T cells	↑ p27^KIP1^ expression Stabilises p27^KIP1^ through transcriptional repression of SKP2 ↓ E2F1 ↓ PKB levels	[73]
	95-D cells	↓ RhoA gene expression and protein levels ↓ MMP-2 ↓ Metastasis and invasion ↑ Cell aggregation	[74]
	GLC cell line	↑ Mitochondrial apoptosis via caspase-dependent and independent manner ↑ Cytochrome *c* and AIF release into the cytoplasm	[75]
	GLC4/ADR cell line	↑ Cytochrome *c* release Activates caspase cascade ↑ PARP cleavage	[76]
*Urothelial*	CNE2 cells	↓ Cell proliferation ↓ Cell migration ↓ Cell invasion Interrupts the cell cycle in G0/G1 phase	[77]
*Nasopharyngeal*	Cisplatin-sensitive or resistant cells J82 253 J T24 RT-112	Blocked autophagy Resensitised cisplatin-resistant cells to apoptotic cell death In combination with cisplatin, prodigiosin sensitised both cisplatin-sensitive and -resistant cell lines to cisplatin ↓ Activities of cathepsin B and L Alters lysosomal function	[22]
*Choriocarcinoma*	JEG3 cell line	↓ IAP family, including XIAP, cIAP-1 and cIAP-2 ↓ Cell growth ↑ Apoptosis ↑ Caspase 3 levels ↑ Caspase 9 levels ↑ PARP cleavage ↑ P53 expression level ↑ Bax/Bcl-2 expression level	[78, 79]
*Prostate cancer*	PC3 cell line PC3 and JEG3 tumour-bearing nude mice	↓ Cell and tumour growth ↑ Bax/Bcl-2 expression level ↑ Apoptosis ↑ PARP cleavage ↑ Caspase 3 levels ↑ Caspase 9 levels ↑ P53 expression level ↓ IAP family, including XIAP, cIAP-1 and cIAP-2	[78]
*Melanoma*	The substrain B16BL6 of mouse melanoma B16 cells	↓ Metastasis and invasion ↑ Mouse survival rate	[74]
	SK-MEL-5 cell line	Activates the mitochondrial apoptotic pathway Disrupts MCL-1/BAK complexes ↓ mTORC1 protein levels ↓ mTORC2 protein levels Loss of AKT phosphorylation	[80, 81]
	SK-MEL-28 cell line	Cell cycle arrest at G0/G1 phase ↑ Apoptosis ↑ DNA damage ↓ Survivin protein levels ↓ Clonogenic capacity in survivin knockdown cells ↓ mTORC1 protein levels ↓ mTORC2 protein levels Loss of AKT phosphorylation	[81, 82]
	SK-Mel-19 cell line	Cell cycle arrest at G0/G1 phase ↑ Apoptosis ↑ DNA damage ↓ Survivin protein levels ↓ Clonogenic capacity in survivin knockdown cells	[82]

**↑**denotes overexpression, upregulation, overactivation, or induction, whereas **↓** expresses reduced activity, suppression, or downregulation

^*^According to the *in-vitro* and *in-vivo* results of an experimental study of prodigiosin-encapsulated scaffolds using blended FDA-approved polymers (polylactic-co-glycolic acid [PLGA], polyethylene glycol [PEG] and polycaprolactone [PCL])

5-FU, 5-fluorouracil; ADR, adriamycin-resistant; AIF, apoptosis-inducing factor; ALL, acute lymphocytic leukaemia; ATP, adenosine triphosphate; ATPase, adenosine triphosphatase; Bax, Bcl-2-associated X protein; Bad, Bcl-2-associated death promoter; Bcl-2, B-cell lymphoma-2; Bak, Bcl2 antagonist/killer; B-CLL, B-Cell chronic lymphocytic leukaemia; BCRP, breast cancer resistance protein; BiP/GRP78, binding immunoglobulin protein-glucose-regulated protein 78; CDK1, cyclin dependent kinase 1; CHOP, C/EBP homologous protein; cIAP-1, cellular inhibitor of apoptosis protein-1; cIAP-2, cellular inhibitor of apoptosis protein-2; CRC, colorectal cancer; CRCSCs, colorectal cancer stem cells; DVL2, dishevelled segment polarity protein 2; E2F1, E2F transcription factor 1; EGFP-LC3, enhanced green fluorescent protein-microtubule-associated protein 1A/1B-light chain 3; EGFR, epidermal growth factor receptor; ER, endoplasmic reticulum; ETC, electron transport chain; GRP78, glucose-regulated protein 78; GSK3β, glycogen synthase kinase 3 beta; HSAEC, human primary small airway epithelial cells; HSP90ɑ, heat shock protein 90 alpha; IAP, inhibitor of apoptosis protein; IRE1–JNK, inositol requiring enzyme 1-c-Jun NH2-terminal kinase; LRP6, low-density lipoprotein receptor-related protein 6; MAPK, mitogen-activated protein kinase; MCL-1/BAK, myeloid-cell leukaemia 1-Bcl2 antagonist/killer MDCK, madindarby canine kidney; MDR1, multidrug resistance 1; MKL, megakaryoblastic leukaemia 1; MMP-2, matrix metalloproteinase-2; MMP-9, matrix metalloproteinase-9; MRP, multidrug resistance-associated protein; MMTV-Wnt1, mice transgenic for mouse mammary tumour virus-Wnt1; mTOR, mammalian target of rapamycin; NAG-1, nonsteroidal anti-inflammatory drug-activated gene-1; PARP, poly (ADP-ribose) polymerase; PERK–eIF2α, protein kinase R (PKR)-like endoplasmic reticulum kinase-eukaryotic translation initiation factor 2A; PI3K, phosphoinositide 3-kinase; PKB, protein kinase B; PUMA, P53 upregulated modulator of apoptosis; ROS, reactive oxygen species; SCLC, small cell lung cancer; SKP2, S-phase kinase associated protein 2; SQSTM, sequestosome; sXBP1, spliced X-box binding protein 1; VEGF, vascular endothelial growth factor; XIAP, X-linked inhibitor of apoptosis

## The possible role of prodigiosin as an ‘immunomodulator’ in cancer

Prodigiosin might improve the efficacy of immunotherapy by regulating multiple immune cells (e.g., T cells) and other proteins in the TME (e.g., programmed death ligand-1 [PD-L1]) [83–87]. Genetic mutations that occur during DNA replication and increased genetic instability in tumours, create neoantigens that evoke an immune response [88]. Failure of immune surveillance facilitates tumour growth and progression despite the expression of immunogenic target expression [89].

### The role of prodigiosin on immune-associated molecules

#### PD-L1

Immune checkpoint inhibitors provide durable clinical response and have become an important anticancer strategy *versus* standard-of-care (SOC) [90]. Targeting PD-1/PD-L1 by antibodies is minimally effective in several cancers, including renal cell carcinoma (RCC) and non-small cell lung cancer (NSCLC) [90, 91]. However, research has reported that failure of immune checkpoint inhibition is attributed to an increased mTOR activity [92]. Inhibition of mTORC1 decreased PD-L1 levels in NSCLC cell lines [93]; although, such inhibition increased PD-L1 levels in other tumour models. For example, everolimus upregulated PD-L1 expression in RCC cell lines and in xenografted tumour tissues [94]. These results indicate that PD-L1 expression levels following mTOR inhibition vary based on tumour types. Hence, using prodigiosin as an effective mTOR inhibitor in different cancer types in vitro and in vivo might explain why mTOR inhibition effects PD-L1 expression levels differently.

#### Heat shock protein 90 (HSP90)

Heat shock proteins are stress hallmarks that are abnormally regulated in cancer to prevent cell degradation and death and preserve the protein structure in a stressful environment [86]. They are essential for the immune system regulatory function in healthy cells; although, cancer cells are drug-resistant due to elevated expression levels of HSP90 [95]. For example, combining bortezomib with HSP90 inhibition improved survival and delayed disease progression in mouse models, and suppressed tumour growth in multiple myeloma (MM) cell culture [96, 97]. Moreover, anti-HSP90 treatment improved T-cell killing in melanoma cell lines, and significantly sustained responses with a better safety profile in relapsed/refractory MM (RRMM) patients [96, 98–101]. Recently, a combination of prodigiosin and the HSP inhibitor, PU-H71, decreased the levels of HSP90α in MDA-MB-231 cells [102]. Among other HSPs, HSP90 stimulates T_regs_ and T helper 1 (Th1) and Th2 cells that support other cells in the immune system. Inhibition of HSP90 using prodigiosin may have the potential to modify T_regs_ and enhance tumour therapy [59].

#### The godfather of tumour suppressors, p53, as a hallmark of inflammation and the immune system function

The P53 protein regulates the immune system and cellular processes, and it is one of the most frequently altered genes that drive malignancy, chemo and radioresistance, and disease progression [103–108]. Wild type *P53* is involved in inflammatory and autoimmune disorders by inducing T_regs_ differentiation. For example, systemic lupus erythematosus (SLE) patients have an inhibited P53 function because the P53 C-terminal domain are bound to autoimmune antibodies [109]. Mice lacking P53 also had autoimmune lesions in liver, lungs, and kidneys. They also had a few number of T_regs_ with impaired differentiation *versus* p53-expressing mice. Additionally, lung- and pancreatic-deficient *P53* tumours exhibited immune tolerance by recruiting both T_regs_ and monocyte/macrophage lineage cells [110].

There may be a functional HSP90─P53 relationship that impacts P53 function, where compounds that disrupt such association would enhance tumour targeting. For example, prodigiosin inhibited HSP90 and rescued P53 in triple-negative breast cancer (TNBC) and P53-deficient CRC cells, respectively, inhibiting tumour growth and leading to tumour cell death [51, 59]. Heat shock protein 90 is a signal protein that controls the function of survivin where HSP90 inhibition dissociates the HSP90-survivin complex, initiating mitochondrial apoptosis and suppressing metastasis [111]. Prodigiosin led to the accumulation of P53, decreased survivin levels, and increased capsase-3 expression levels in chemoresistant acute lymphoblastic leukaemia (ALL) [112]. Notably, prodigiosin initiates selective apoptosis in malignant breast cancer cell lines regardless of MDR or the P53 status [44, 113]. In contrast, prodigiosin did not accumulate P53 in human CLL cells *versus* doxorubicin-treated cells [68].

### The role of prodigiosin on immune cells in the TME

#### T_regs_

Cancer immunotherapy relies partly on the immunomodulating actions mediated by conventional (T_conv_) T cells and T_regs_ [114]. Regulatory T cells maintain the proper function of the adaptive immune system; however, they can also suppress anticancer immunity and lead to poor disease prognosis (e.g., NSCLC, breast cancer) [115–118]. Patients at high risk of ovarian and breast carcinomas are expected to have reduced survival rates due to elevated levels of T_regs_ [119, 120]. Heat shock proteins facilitate the immunosuppressive function, division and growth, and cytokine release of T_regs_ [99]. A second-generation HSP90 inhibitor (i.e., ganetespib) reduced the number of T_regs_ in skin cancer in vitro and in vivo. Similarly, the notion that prodigiosin inhibited HSP90α expression levels in TNBC cells supports that it may prevent T_regs_-mediated immunosuppression in the TME. Prodigiosin may also decrease T_regs_ numbers and enhance their antitumour functions by suppressing HSP90 and survivin as well as activating p53 simultaneously. Moreover, prodigiosin may modulate T_regs_ differentiation and prevent immune tolerance because of its effect on P53 irrespective of its status.

#### T lymphocytes

Despite their antigen-directed cancer cytotoxicity, overstimulation of T cells (i.e., T cell exhaustion) causes T cell senescence with defects in effector functions and proliferation, preventing tumour control [121–123]. Persistent antigen exposure helps tumours evade the immune surveillance and causes T cell dysfunction, where dysfunctional T cells have multiple inhibitory receptors such as PD-1 [124]. Prodigiosin selectively suppressed the proliferation and immune functions of T cells but not B-cells in vitro and in vivo [125]. However, data are insufficient to confirm whether prodigiosin directly or indirectly inhibited the immune functions of T cells. Prodigiosin also suppressed IL-2Rα expression in the IL-2/IL-2R signalling to block T-cell activation, inhibiting graft *versus* host disease (GvHD) and delayed the progression of autoimmune diabetes without toxicity in mice [126]. Prodigiosin 25-C (a related compound) directly attacked the activated CD8^+^ T cells by inhibiting the acidification of intracellular organelles needed for cytotoxic T lymphocytes (CTLs) functions [127]. Prodigiosin represents an effective molecule in an immunosuppressive TME characterised by dysfunctional T cells, and might be an important molecule for immunologic studies on T cells [126]. Studying the extent of T-cell inhibition after treatment with prodigiosin is noteworthy, because deficient T-cell inhibition causes autoimmune diseases, whereas cancers arise due to excessive T-cell inhibition [128].

#### B lymphocytes

Regardless of the available consensus about the immunosuppressive role of prodigiosin on T-cell proliferation, little is known about its effects on B cells [129, 130]. B cells constitute ~ 25% of all cells in some cancers and 40% of tumour-infiltrating lymphocytes (TILs) in breast cancer patients [131–133]. B cells destruct tumours by increasing T cell responses and support tumour growth by favouring immunosuppression via complement activation or immune complex formation [134]. Prodigiosin inhibited polyclonal B-cell proliferation and immortalisation in human peripheral blood lymphocytes (PBLs) and Epstein Barr virus (EBV) [130]. The differential response of prodigiosin on T and B cells might be attributed to the source of the cells used in the experiments. For instance, human cells demonstrate selective inhibition of T-cell proliferation compared to mouse cells [23, 129, 130, 135]. Moreover, B cells are heterogeneous and diverse, and might increase T-cell anticancer activities or facilitate carcinogenesis through angiogenesis, inflammation, and immunosuppression [134].

#### Tumour-associated macrophages (TAMs)

**Fig. 3:**
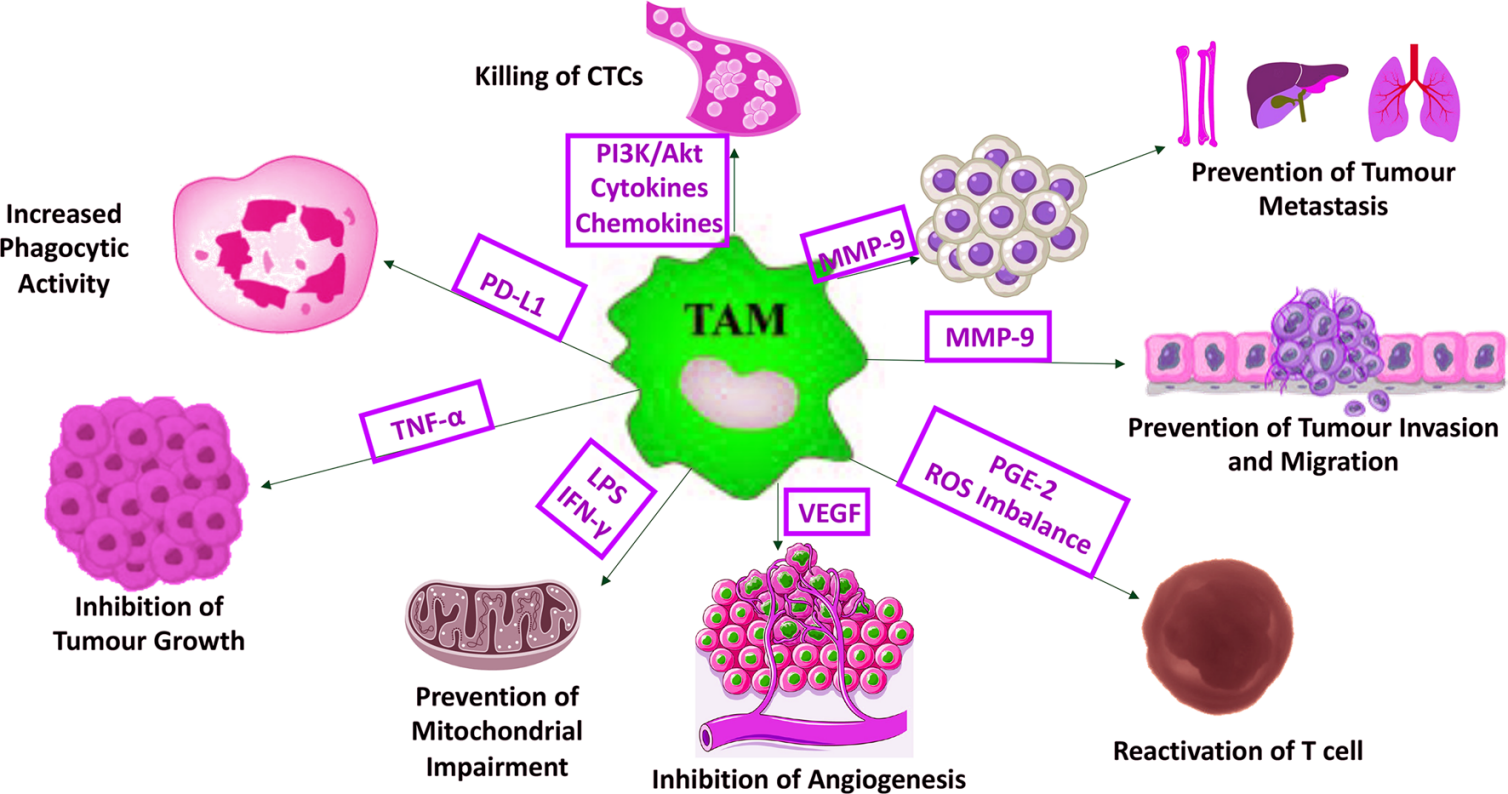
A proposed model for the immunomodulatory effect of prodigiosin on TAM-mediated immunosuppression via ROS imbalance and inhibition of MMP-9, PGE-2, VEGF, TNF-α, PI3K/Akt, LPS, IFN-γ, cytokines, and chemokines. CTCs, circulating tumour cells; IFN-γ, interferon-gamma; LPS, lipopolysaccharide; MMP-9, matrix metalloproteinase-9; PD-L1, programmed death-ligand 1; PGE-2, prostaglandin E2; PI3K/Akt, phosphoinositide-3-kinase–protein kinase/Akt; ROS, reactive oxygen species; TAM, tumour-associated macrophages; TNF-α, tumour necrosis factor-alpha; VEGF, vascular endothelial growth factor

Existing data support the interesting role of prodigiosin in modulating tumour-associated macrophages (TAMs). Recruited macrophages into the TME are converted into TAMs, certain types of immunosuppressive macrophages that promote “tumour tolerance” by suppressing the generation and function of antitumour T cells [136, 137]. Solid tumours (e.g., breast, prostate) had accumulations of TAMs that confer poor disease prognosis [138–140]. Prodigiosin is proapoptotic and effective against both epidermal growth factor receptor (EGFR) and vascular endothelial growth factor (VEGF). It might also prevent the growth of malignant tumours by inhibiting the induction of TAM infiltration and M2 polarisation (Fig. 3) [102, 141, 142]. For example, less M2-polarised TAMs exist in CRC mouse models after EGFR signal disruption by gene knockout (KO) or cetuximab [18, 143]. Involvement of the phosphatidylinositol 3-kinase (PI3K/Akt) pathway in TAM regulation and the inhibitory effect of prodigiosin on this pathway, suggest that prodigiosin might prevent TAM recruitment and initiate tumour-necrosis factor (TNF)-related apoptosis [26, 144–146]. TAMs-induced matrix metalloproteinase-9 (MMP-9) and VEGF also mediate metastasis in a TNBC mouse model and primary lung cancer tissues [147, 148]. Prodigiosin inhibited matrix metalloproteinase-9 (MMP-9) that is argued to release VEGF to regulate TAM-driven tumour growth and angiogenesis (Fig. 3) [67, 149–152].

Prodigiosin might interfere with TAMs-secreted nicotinamide adenine dinucleotide phosphate oxidase (NOX) function or suppress its upregulation in the TME, preventing oxidative stress-induced carcinogenesis. Tumour-associated macrophages secrete chemokines and cytokines (e.g., interleukins [ILs], prostaglandin E2 [PGE2], and TNF-α) that facilitate carcinogenesis, and express NOX2 that maintains immunological tolerance, tumourigenesis, and metastasis [153–156]. Prodigiosin analogue inhibited NOX activation by affecting the translocations of p47^phox^ and Rac protein to the plasma membrane in a mouse macrophage cell line [157]. Targeting NOX2 by prodigiosin to reduce metastasis warrants further investigation, considering reactive oxygen species (ROS) source, tumour cells’ susceptibility to ROS toxicity, cancer progression stage, and effector cells’ sensitivity to ROS‐induced immunosuppression.

Prodigiosin might modulate the immune response of TAMs by inhibiting TNF-α, IL-2, and interferon-gamma (IFN-γ), reducing TAMs-mediated immunosuppression. Cuevas et al*.,* recently showed that prodigiosin modulated the immune response and stabilised atherosclerotic lesions by inhibiting circulating TNF-α, IL-2, and IFN-γ in vivo (Fig. 3) [158]. Activation of M1 macrophages via IFN-γ is essential in immune function and contributes to tissue damage by proinflammatory cytokines [136]. For example, IFN-γ switched the immunosuppressive TAMs into immunostimulatory cells, potentiating the efficacy of antitumour immunotherapies by generating effector T cells in ovarian cancer [137]. Nevertheless, IFN-γ also conditioned protumourigenic effects in solid tumours and induced lung colonisation and enhanced expression of class I major histocompatibility complex (MHC I)-related antigens [159, 160]. Prodigiosin also inhibited the onset and progression of autoimmune diabetes in non-obese diabetic mice, and reduced IL-2, IFN-γ, and TNF-α mRNA levels in prodigiosin-treated group without side effects [161]. Nonetheless, prodigiosin did not inhibit the secretion of IL-2 in vitro but inhibited the mitogenic signalling from IL-2, suggesting an unusual mechanism of action [135]. It is important to consider the negative effect of prodigiosin on IL-2, because it is among the most potent inducers of antitumour activity in preclinical studies [162].

**Fig. 4:**
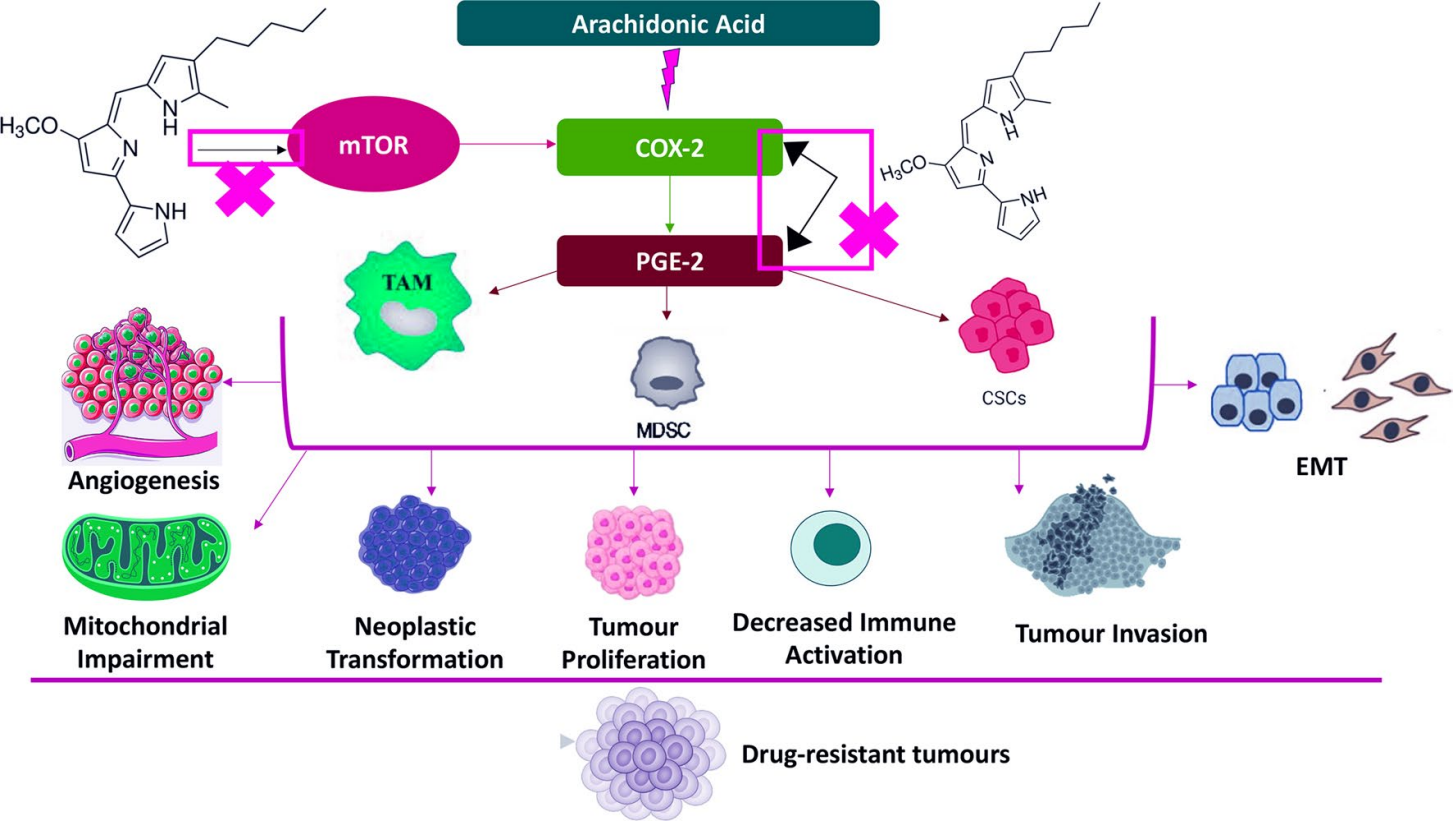
The inhibitory effect of prodigiosin on mTOR and COX-2/PGE-2 pathways that further sensitise tumour cells to drugs. COX-2, cyclooxygenase-2; CSCs, cancer stem cells; EMT, epithelial-mesenchymal transition; PGE-2, prostaglandin E2; MDSCs, myeloid-derived suppressor cells; mTOR, mammalian target of rapamycin; TAM, tumour-associated macrophages

Solid tumours are characterised by suppressed antitumour immunity due to high PGE2 levels that reduce apoptosis, and increase tumour growth, invasion, metastasis, and angiogenesis [163–166]. There is a TAMs-PGE2 reciprocal relationship where TAMs secrete PGE2 that directly inhibits CD4^+^ and CD8^+^ T cells’ effector function, while PGE2 regulates macrophage polarisation into M2 TAMs [166–168]. Activation of the COX-2/PGE2 pathway also stimulates PD-L1 expression via TAMs to inhibit the immune response and promote immune tolerance by modulating T-cell activity and facilitating cancer immune escape [155, 169]. Accordingly, it is wise to consider that prodigiosin-related mTOR inhibition may interfere indirectly with the COX-2/PGE2 pathway by decreasing PD-L1 levels (Fig. 4) [93]. However, mTOR inhibition simultaneously upregulates PD-L1 expression in some circumstances such as in xenografted tumour tissues and in RCC cell lines [94].

#### Tumour-associated dendritic cells (TADCs)

Similar to TAMs, prodigiosin might modulate TADCs immune functions via PGE2. Although DCs initiate T-cell anticancer immune response, malignant tumours possess other types of DCs with reduced migration and accumulation in lymphoid organs that lead to immunosuppressive T cells [170]. High PGE2 levels shift the immunostimulatory DCs into immunosuppressive cells to reduce the proliferation of anticancer T cells by upregulating PD-L1 [170]. Prostaglandin E2 inhibits MHC II expression and upregulates IL-10 via EP2 and EP4 receptors, suppressing DCs’ antigen presentation mediated via the COX-2/EP3 signalling [171, 172]. The immunomodulatory actions of prodigiosin on TAMs discussed earlier denote that it may affect DCs in the TME. Prodigiosin might reverse TAM-mediated attenuation of tumouricidal and tumour antigen-presenting behaviours occurring to DCs due to the established metabolic crosstalk [142].

#### NK cells

**Fig. 5:**
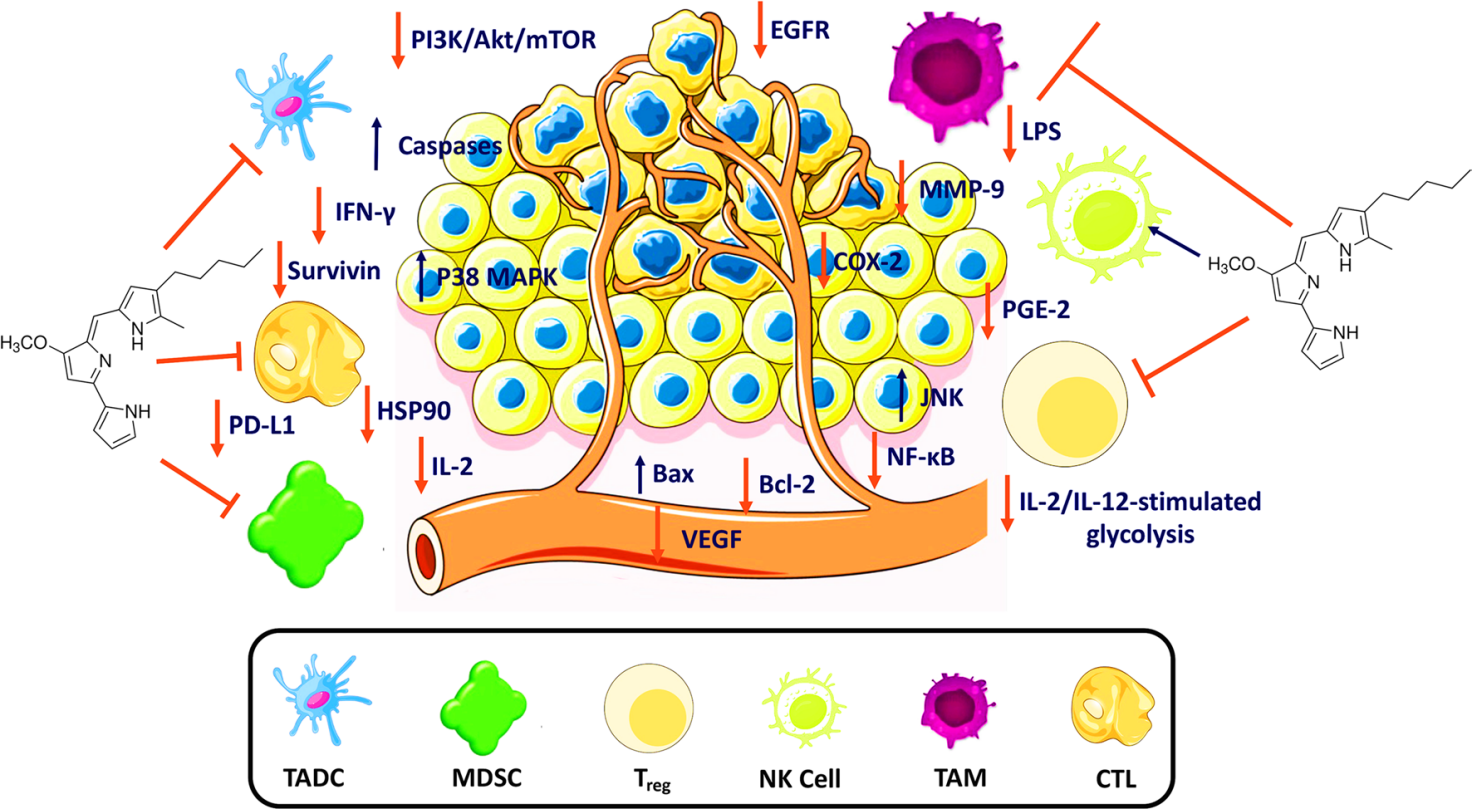
The potential role of prodigiosin as an immunomodulator in the TME. COX-2, cyclooxygenase-2; CTL, cytotoxic T lymphocyte; HSP90, heat shock protein 90; IFN-γ, interferon-gamma; IL, interleukin; JNK, c-Jun N-terminal kinases; LPS, lipopolysaccharide; MAPK; mitogen-activated protein kinase; MDSC, myeloid-derived suppressor cell; MMP-9, matrix metalloproteinase-9; mTOR, mammalian target of rapamycin; NF-κB, nuclear factor kappa B; NK, natural killer; PD-L1, programmed death-ligand 1; PGE-2, prostaglandin E2; PI3K/Akt, phosphoinositide-3-kinase–protein kinase/Akt; TADC, tumour-associated dendritic cell; TAM, tumour-associated macrophage; T_reg_; regulatory T cell; VEGF, vascular endothelial growth factor

Activated NK cells eliminate tumours via death receptor-mediated killing, granule exocytosis, and cytokine production (i.e., IFN-γ) that stimulates other immune cells [173]. Nevertheless, PGE2 per se trades off NK cell activities (e.g., tumour lysis) for metastases development via activated EP2 and EP4 receptors [171, 174]. Prostaglandin E2 suppresses the function of NK cells by multiple mechanisms, such as inhibiting IFN-γ production and ILs–induced IFN-γ expression in NK cells via EP2 receptor or downregulating NK receptors through the cAMP/PKA pathway [171]. EP4 antagonist inhibited PGE2-mediated NK cell suppression by protecting IFN-γ production by NK cells, inhibiting breast and lung tumour metastases [175–177]. Prodigiosin might have an immunomodulatory role since the reciprocal NK-DCs crosstalk is inhibited by PGE2 through chemokine and cytokine modulation (Fig. 5) [178].

#### MDSCs

Tumours maintain an immunosuppressive TME through high levels of heterogeneous immature myeloid cells, referred to as MDSCs [179]. It is mandatory to target myeloid populations that stop anticancer immunity or activate stimulatory cells that promote antitumour immunity. In addition to ILs and VEGF, PGE2-rich tumoural exosomes induce MDSCs activation and migration and promote MDSCs-dependent tumour growth [179–182]. Prostaglandin E2 controls MDSCs differentiation and increases their levels, enhancing the stemness of cervical cancer cells in vitro and in vivo (Fig. 4). For example, MDSCs express the four PGE2 receptors (i.e., EP1-4) in tumour-bearing mice [183, 184].

COX-2 inhibitors reduce MDSCs levels and delay the burden of primary carcinoma tumour, because PGE2-induced COX-2 activates the secretion of endogenous MDSCs-related PGE2 (Fig. 4) [185]. Production of PGE2 in lung and ovarian cancers is correlated with COX-2 expression, promoting recruitment and retention of MDSCs [185, 186]. COX-2 inhibitors reduce PGE2 production resulting in decreased levels of MDSC-attracting C-C Motif Chemokine Ligand 2 (CCL2) in vivo, suggesting that blocking COX-2 impedes the development and accumulation of MDSCs [187]. *In–silico* molecular docking analysis revealed that prodigiosin inhibited COX-2 effectively and could be assessed as an antiinflammatory compound in further research [188]. Inflammation is characterised by high levels of PGE2 through COX-2. Moreover, overexpression of COX-2 (also a downstream target of mTORC1) promoted proliferation and growth of several cancers. Downregulation of COX-2 exerts a protective effect against hyperactivated mTORC1-mediated tumourigenesis caused by loss of tuberous sclerosis complex (TSC) in TSC-null cell [189]. Likewise, the ability of prodigiosin to inhibit the mTOR pathway might prevent COX-2-mediated tumourigenesis via increased PGE2 production (Fig. 4).

Production of PGE2 by MDSCs increases PD-L1 expression in ovarian cancer through the mTOR signalling pathway (Fig. 4). Bone marrow (BM) cells cultured with bladder cancer cells showed significant PD-L1 expression in monocytic MDSCs [183]. Tumour-infiltrating PD-L1^+^ cells also showed high expression levels of both COX-2 and PGE2 synthase 1 (mPGES1) in tumour-bearing mice. Inhibition of mPGES1/COX-2 by prodigiosin may reduce the expression of MDSCs-related PD-L1, arguing that reprogramming PGE2 metabolism enhances tumour sensitivity to immunotherapy.

#### The role of prodigiosin in the metabolic reprograming of immune cells

The interesting anticancer and immunomodulatory actions of prodigiosin (Table 1) prompt us to further discuss whether it is involved in the metabolic reprogramming of TME-related immune cells (Fig. 5) [190]. Interaction of the principal metabolic pathways in immune cells (e.g., pentose phosphate pathway [PPP], fatty acid oxidation [FAO], Krebs’ cycle, and glycolysis), provides energy and nutrients to maintain their activity. Both metabolites and metabolic activity regulate autophagy, apoptosis, and posttranslational modifications, and pro and antiinflammatory effects [191–194].

##### NK cells

The mTOR/PI3K pathway is sensitive to a high number of extracellular signals and is a key regulator of cellular growth, proliferation, and metabolism. Cancer is characterised by an aberrant mTOR signalling that supports tumour proliferation, survival, metabolic programming, and drug resistance [195]. The mTOR signalling pathway enhances glycolysis and mitochondrial function to regulate the metabolism of NK cells. Inhibition of mTOR by rapamycin reduced both IL-2/IL-12-stimulated glycolysis and IL-2-stimulated levels in mouse NK cells and human NK cell glycolysis, respectively (Fig. 5) [196, 197]. For instance, higher glucose transporter 1 (GLUT1) levels that absorb glucose exist following the upregulation of both CD71 and CD98 [198, 199]. The anti-mTOR activity of prodigiosin in Table 1 highlight that it might have a pivotal role in the metabolic reprogramming of NK cells [59].

##### TAMs

Macrophages with different polarisation states are also different in glycometabolism. Anaerobic glycolysis provides instant energy to help proinflammatory M1 macrophages eliminate pathogens, whereas mitochondrial oxidative phosphorylation (OXPHOS) generates energy for antiinflammatory M2 macrophages [200]. Therefore, mitochondrial dysfunction impedes M2 repolarisation that inhibits regulatory immune signals, and facilitates tumour angiogenesis, migration, and metastasis [166, 201].

Prodigiosin suppresses inflammatory responses induced by lipopolysaccharide (LPS) in activated murine macrophage, by inhibiting the activation of p38 mitogen-activated kinase (MPAK), c-Jun N-terminal kinase (JNK), and nuclear factor kappa B (NF-κB) (Fig. 5). Stimulation of LPS and IFN-γ reduces OXPHOS levels, impairing the mitochondrial function required for M2 repolarisation with the accumulation of hypoxia-inducible factor-1α (HIF-1α) and metabolites of Krebs’ cycle (e.g., succinate) [202]. Hypoxia-inducible factor-1α regulates GLUT1 to affect the polarisation and functions of macrophages via metabolic reprogramming of PPP and anaerobic glycolysis [203]. In this regard, prodigiosin might prevent mitochondrial impairment (Fig. 3) and facilitate ‘M2 polarisation’. Namely, inhibition of LPS and IFN-γ increases OXPHOS levels (Figs. 3, 5), implicating a mechanism by which prodigiosin be immunosuppressive where M2 macrophages prevail to favour tumour progression and tissue repair.

##### T lymphocytes

Naïve T cells preserve a resting state using OXPHOS in contrast to activated T cells that grow via glucose and lipid metabolism. Proliferating cells exhibit higher aerobic glycolysis rate, referred to as the Warburg effect where the principal driver of aerobic glycolysis is ‘mitochondrial dysfunction’ [204, 205]. Imbalance of ROS production due to mitochondrial dysfunction destroys cell membranes and DNA, disrupts cell proliferation, induces apoptosis, and inhibits autophagy [206–210]. Prodigiosin fosters the protecting antioxidative function of nuclear factor erythroid 2-related factor 2 (Nrf2) and scavenges ROS in hepatocellular carcinoma (HCC) cells [211, 212]. However, it upregulates ROS levels and suppressed proliferation and autophagy in leukaemia cell line. These data suggest that prodigiosin might interfere with be the metabolic reprogramming of T cells via ROS, considering its ROS stimulatory and scavenging roles (Fig. 3).

The mTOR pathway is crucial in upregulating GLUT1 expression in naïve T cells to promote glucose absorption and to improve the immune response [213–215]. It also helps Th2 cells’ differentiation via the OXPHOS–aerobic glycolysis metabolic transition. Mammalian target of rapamycin complex 1 (mTORC1) is essential for Th1 cells while mammalian target of rapamycin complex 2 (mTORC2) regulates OXPHOS and glycolysis in Th2 cells [216–218]. Moreover, the mTOR pathway regulates the production and memory differentiation of CD8^+^ T cells [219–223]. Consistent with the inhibition of the PI3K/Akt/mTOR pathway by prodigiosin in cancer, it might be involved in the metabolic reprogramming of Th cells and CD8^+^ T cells [42, 48, 71, 72]. However, these data should be used cautiously because the ability of prodigiosin to suppress the immune functions of T cells has not been confirmed yet.

##### TADCs

Metabolic reprogramming (i.e., decreased OXPHOS and an increased glycolysis) is essential for activation and functions of DCs [224]. Stimulated LPS helps DCs regulate the mTOR signal, stabilise HIF1-α, and increase inducible nitric oxide synthase (iNOS) expression [225, 226]. Since prodigiosin inhibits the mTOR pathway and reduces iNOS expression by inhibiting LPS-triggered inflammatory responses, it might prevent the manipulation of the metabolic processes that affect the activation and functions of DCs (Fig. 5) [72, 227]. However, the metabolic environment where DCs compete with neighbouring cells for nutrition is difficult to simulate and measure both in vitro and in vivo [203].

### Given these, does prodigiosin have possible immunomodulatory actions on TAMs, TADCs, NK cells, and MDSCs via COX-2/PGE2 in cancer?

The crosstalk between immune cells outlines the potential of prodigiosin to interfere with the COX-2/PGE2 pathway (Fig. 4) and decrease the immunological tolerance mediated by TAMs, TADCs, NK cells, and MDSCs. Nonetheless, the opposite effects of PGE2 (suppressive/protective) due to disease course (e.g., cancer, gastric lesions) and status of the immune system, should be considered while developing cancer-specific treatments. For instance, PGE2 protected rats from HCl/ethanol-induced gastric lesions by reducing the levels of antioxidants, apoptotic biomarkers, and inflammatory mediators [228]. Prodigiosin prevented apoptosis in the gastric mucosa by downregulating the expression of COX-2, caspase-3, IL1-β, Bax, and TNF-α, while upregulating Bcl-2 expression; hence, increasing PGE2 production [228]. However, these conditions favour tumour growth and proliferation. These data are confirmed by the idea that reducing PGE2 levels might prevent tumour initiation, inhibit tumour growth and metastasis, reprogram antitumour immunity, and increase the efficacy of immunotherapies.

#### Tumour mutation burden (TMB)

Analysis of ~ 5000 mutations from ~ 7000 cancers highlighted that tumour mutation burden (TMB) status in cancer cells—the number of somatic mutations/megabase of the genome encoding tumours—successfully predicted the efficacy of immune checkpoint blockade [229]. Studies demonstrated that PI3K/mTOR pathway mutations are correlated with TMB status in NSCLC and nasopharyngeal carcinoma [230, 231]. Treatment with an mTOR inhibitor (i.e., everolimus) led to tumour shrinkage and disease stabilisation in patients with NSCLC [230]. Prodigiosin may have an interesting role on TMB by inhibiting PI3K/mTOR pathway and P53 (Table 1) [50].

## Conclusion

The current review demonstrated the compelling immunomodulatory and metabolic reprogramming activities of prodigiosin on TME-related immune cells (Fig. 5). Particularly, the crosstalk between the immune cells, the involvement of the mTOR pathway, and expression of PGE-2 and COX-2, dictate the potential of prodigiosin as an immunomodulator in the TME. Using prodigiosin to inhibit the PI3K/mTOR pathway may clarify its hypothesised effects on TMB, especially because the TMB status associated with PI3K/mTOR pathway gene mutations is still unclear.

### Future perspectives

Further research may confirm whether prodigiosin has a compensatory mechanism that overcomes cancer resistance, and whether it renders B cells pro or antitumourigenic. More data are required to examine the controversial role of prodigiosin in blocking T-cell activation by suppressing IL-2Rα expression in the IL-2/IL-2R signalling. It is also important to focus on prodigiosin encapsulation in nanoparticles because limited research demonstrated that it might be an excellent alternative in cancer treatment. Based on the crosstalk between immune cells, using prodigiosin in cancer immunotherapy elicits outstanding inquiries such as:

- Could prodigiosin be used as a tool to identify the point at which immune tolerance occurs?
- What is the dominant role (immunostimulatory/immunosuppressive) of prodigiosin on every immune cell in the TME?
- How could prodigiosin modulate NK-DC crosstalk and strengthen both DC- and NK-cell-mediated immune response?

## Data Availability

All data generated or analysed during this study are included in this published article.

## Abbreviations

ALL: Acute lymphoblastic leukaemia
ATRA: All-trans retinoic acid
CLL: Chronic lymphoblastic leukaemia
COX: Cyclooxygenase
CRC: Colorectal cancer
CTLs: Cytotoxic T lymphocytes
CVD: Cardiovascular disease
EBV: Epstein Barr virus
EGCG: Epigallocatechin gallate
EGFR: Epidermal growth factor receptor
FAO: Fatty acid oxidation
GLOBOCAN: Global burden of cancer
GLUT1: Glucose transporter 1
GvHD: Graft versus host disease
HCC: Hepatocellular carcinoma
HIF-1α: Hypoxia-inducible factor-1α
HSP90: Heat shock protein 90
IFN: Interferon
ILs: Interleukins
iNOS: Inducible nitric oxide synthase
JNK: C-Jun N-terminal kinase
KO: Knock out
LPS: Lipopolysaccharide
MDR: Multidrug resistance
MDSCs: Myeloid-derived suppressor cells
MHCI: Class I major histocompatibility complex
MM: Multiple myeloma
MAPK: P38 Mitogen-activated kinase
mTOR: Mammalian target of rapamycin
NF-κB: Nuclear factor kappa B
NK: Natural killer
NOX: Nicotinamide adenine dinucleotide phosphate oxidase
NSCLC: Non-small cell lung cancer
OXPHOS: Oxidative phosphorylation
PBLs: Peripheral blood lymphocytes
PD-1: Programmed death protein 1
PD-L1: Programmed death ligand 1
PGE2: Prostaglandin E2
PGES1: Prostaglandin E2 synthase 1
PI3K: Phosphatidylinositol 3-kinase
PPP: Pentose phosphate pathway
PSK: Polysaccharide K
RCC: Renal cell carcinoma
ROS: Reactive oxygen species
RRMM: Relapsed/refractory multiple myeloma
SLE: Systemic lupus erythematosus
SOC: Standard-of-care
TADCs: Tumour-associated dendritic cells
TAMs: Tumour-associated macrophages
TILs: Tumour-infiltrating lymphocytes
TMB: Tumour mutation burden
TME: Tumour microenvironment
TNBC: Triple-negative breast cancer
TNF: Tumour-necrosis factor
T_regs_: Regulatory T cells
TSC: Tuberous sclerosis complex
VEGF: Vascular endothelial growth factor
WHO: World Health Organisation
